# Switching from adalimumab to tofacitinib in the treatment of patients with rheumatoid arthritis

**DOI:** 10.1186/s13075-016-1049-3

**Published:** 2016-06-23

**Authors:** Mark C. Genovese, Ronald F. van Vollenhoven, Bethanie Wilkinson, Lisy Wang, Samuel H. Zwillich, David Gruben, Pinaki Biswas, Richard Riese, Liza Takiya, Thomas V. Jones

**Affiliations:** Division of Rheumatology, Stanford University, Palo Alto, CA USA; Unit for Clinical Therapy Research, Inflammatory Diseases, Karolinska Institute, Stockholm, Sweden; Pfizer Inc, Groton, CT USA; Pfizer Inc, 500 Arcola Drive, F5352, Collegeville, PA 19426 USA

**Keywords:** Drug switching, Efficacy, Safety, Tofacitinib, Adalimumab, Rheumatoid arthritis

## Abstract

**Background:**

Tofacitinib is an oral Janus kinase inhibitor for the treatment of rheumatoid arthritis (RA). The aim of this study was to explore the safety and efficacy of open-label tofacitinib following blinded treatment with adalimumab or tofacitinib for moderate to severe RA.

**Methods:**

Analyses included patients treated with adalimumab 40 mg once every 2 weeks or tofacitinib 10 mg twice daily (BID) with background methotrexate (MTX) in a 12-month randomized study (NCT00853385), who subsequently received tofacitinib 10 mg BID (with/without background MTX) in an open-label extension (NCT00413699). Patients with treatment-related serious adverse events (AEs) and serious or recurrent infections in the index study were excluded from the extension study. Exposure-adjusted incidence rates of safety-related events were assessed in 3-month and 12-month periods in the year before and in the year after switching. Efficacy was assessed 3 months before, at the time of, and 3 months after switching.

**Results:**

There were 233 (107 adalimumab to tofacitinib 10 mg BID, 126 blinded to open-label tofacitinib 10 mg BID) patients included in these analyses. Patients in both treatment sequences had similar incidence rates (per 100 patient-years) of discontinuation due to AEs, serious AEs, and serious infections in the year before and in the year after switching. Incidence rates of AEs were increased in the first 3 months after switching compared with the last 3 months before switching in both treatment groups. Switching from either blinded adalimumab or tofacitinib to open-label tofacitinib resulted in numerically higher incidence of responders for signs and symptoms of disease and improved physical function.

**Conclusions:**

Treatment can be directly switched from adalimumab to tofacitinib. A similar safety and efficacy profile was seen when patients received open-label tofacitinib after receiving either blinded adalimumab or tofacitinib.

**Trial registration:**

ClinicalTrials.gov Identifiers: NCT00853385, registered 27 February 2009; NCT00413699, registered 18 December 2006.

**Electronic supplementary material:**

The online version of this article (doi:10.1186/s13075-016-1049-3) contains supplementary material, which is available to authorized users.

## Background

The long-term treatment of rheumatoid arthritis (RA) often involves a sequence of different therapies. Patients who have not responded to conventional synthetic disease-modifying anti-rheumatic drugs (DMARDs) have been routinely treated with a tumor necrosis factor inhibitor (TNFi); however, approximately 20 % of patients discontinue TNFi therapy within 12 months due to inefficacy or adverse events (AEs) [1, 2]. Management of patients who have not responded to treatment with one TNFi may involve switching to another TNFi or an agent with an alternative mechanism of action. Several randomized controlled trials have demonstrated the efficacy of agents with a different mechanism of action versus placebo in patients with an inadequate response to a TNFi [3–5]. In clinical practice, patients receiving a TNFi, such as adalimumab (ADA), may need or choose to switch to an agent with an alternative mechanism of action; an understanding of the impact of switching without an extended washout period is needed.

Tofacitinib is an oral Janus kinase inhibitor for the treatment of RA [6]. The tofacitinib phase 3 program included six randomized, controlled studies in which tofacitinib was assessed in adult patients with active moderate to severe RA [7–12] as monotherapy or combined with conventional synthetic DMARDs. A 12-month, double-blind, randomized, controlled trial, conducted in 115 centers worldwide, compared the safety and efficacy of tofacitinib 5 or 10 mg twice daily (BID) or ADA 40 mg every 2 weeks (Q2W) with placebo in patients with active RA taking background methotrexate (MTX; 7.5 to 25 mg weekly), who had an inadequate response to MTX (defined as sufficient residual disease activity to meet entry criteria). The primary safety and efficacy data have been reported previously [10]. Eligible patients who had participated in this blinded study could be enrolled in an open-label extension where they received tofacitinib 10 mg BID (with or without background MTX), regardless of their treatment in the blinded study [13].

The objective of the current analyses was to describe the safety and efficacy of open-label tofacitinib in an extension study, following a direct switch from blinded treatment with either ADA or tofacitinib for the treatment of moderate to severe RA. The group of patients who received blinded tofacitinib followed by open-label tofacitinib in the extension study could act as a control for such an assessment by representing potential changes to the clinical profile introduced by transition from the double-blind to the open-label study with minimal changes in treatment (dose adjustments permitted for concomitant RA medications and tofacitinib in the extension study).

## Methods

### Study design and treatment

ORAL Standard (A3921064; NCT00853385) was a double-blind, phase 3 trial [10] in which patients were randomly assigned to one of five regimens, all with stable background MTX: tofacitinib 5 mg BID, tofacitinib 10 mg BID, ADA 40 mg Q2W (subcutaneous injection), placebo for 3 or 6 months followed by tofacitinib 5 mg BID, and placebo for 3 or 6 months followed by tofacitinib 10 mg BID for a total of 12 months.

ORAL Sequel (A3921024; NCT00413699) is an open-label long-term extension study [13] in which all patients from ORAL Standard were eligible to receive tofacitinib 10 mg BID with or without conventional synthetic DMARDs. Exclusion criteria included treatment-related serious AEs and serious or recurrent infections (including serious or recurrent herpes zoster) in the index study. Patients with latent tuberculosis were allowed to continue in the extension study if they had received an adequate course of therapy. Patients from certain countries (e.g., Korea, Croatia, Denmark, Czech Republic, Germany, Spain, Sweden, Ireland and the United Kingdom) who enrolled in the extension study ≥14 days after their final index study visit were excluded if their absolute lymphocyte count (ALC) values were <750 cells/mm^3^. Patients from the remaining countries were excluded if their ALC values were <500 cells/mm^3^.

The current post-hoc analyses included patients who were randomized at baseline to treatment with blinded ADA 40 mg Q2W + MTX or blinded tofacitinib 10 mg BID + MTX in ORAL Standard and subsequently received open-label tofacitinib 10 mg BID (with or without MTX) in the extension study. Although the patients initiated treatment in the long-term extension with tofacitinib 10 mg BID, this could be reduced to 5 mg BID for safety reasons. Additionally, dose adjustments, including tapering and discontinuation (DC) of permitted concomitant RA medications (such as MTX, prednisone and non-steroidal anti-inflammatory drugs) were allowed in response to inadequate efficacy, disease improvement, or for safety reasons.

Each study was performed in compliance with the Declaration of Helsinki and Good Clinical Practice Guidelines established by the International Conference on Harmonization. The final protocols, amendments, and informed consent documentation were reviewed and approved by the institutional review board and the independent ethics committee of each investigational center (Additional file 1: Table S1).

### Patients

Patients in this analysis included those who were initially enrolled in the phase 3, ORAL Standard study [10] and subsequently elected to enroll in the extension study [13]. Eligible patients in the phase 3 study were ≥18 years of age and had received a diagnosis of active RA, as classified according to the American College of Rheumatology (ACR) 1987 Revised Criteria [14] and had not failed or had an AE to a TNFi in the past. Additional inclusion and exclusion criteria have been described previously [10]. Patients who completed participation in the study or discontinued for reasons other than treatment-related serious AEs and who met other inclusion/exclusion criteria were invited to enroll in the long-term extension [13]. For patients enrolling in the long-term extension study within 2 weeks of their last dose in the blinded study, baseline values were obtained from the blinded study.

### Statistical analyses

Post-hoc descriptive statistics are reported (data cutoff date 19 April 2012). As the extension study is ongoing, the study database has not yet been locked (i.e., some values may change for the final, locked study database).

#### Safety and tolerability assessments

The incidence of treatment-emergent AEs (TEAEs) was calculated at scheduled visits of the blinded study (baseline, and months 1, 3, 6, 9, and 12) and extension study (baseline, months 1, 2, 3, and every 3 months thereafter).

In the current analyses, the incidence of TEAEs was compared during the last 3 months of blinded therapy and the first 3 months of open-label treatment.

Exposure-adjusted incidence rates (and corresponding 95 % confidence intervals) of DCs due to AEs, serious AEs and serious infections per 100 patient-years from baseline were calculated for patients who completed treatment with ADA 40 mg Q2W + MTX or tofacitinib 10 mg BID + MTX in the blinded study for patients, (1) regardless of whether they entered the extension study, and (2) who then enrolled in the extension study. These analyses were performed for 3-month (months 0 − 3, 3 − 6, 6 − 9, and 9 − 12) and 12-month (months 0 − 12) periods for both the blinded study and the first 12 months of the extension study. Of note, there were two study visits in the last 3 months of the blinded study (visits 5 and 6 at months 9 and 12, respectively), and four study visits in the first 3 months of the extension study (visits 1 − 4, for baseline and months 1, 2, and 3).

#### Efficacy assessments

The following efficacy parameters were measured in patients who completed treatment with ADA + MTX or tofacitinib 10 mg + MTX in the blinded study (or discontinued for reasons other than a treatment-related serious AE) and then switched to the tofacitinib 10 mg group in the extension study: ACR response rates (20 %, 50 %, and 70 % improvement), mean change in Health Assessment Questionnaire-Disability Index (HAQ-DI) scores from the baseline of the blinded study, and Disease Activity Score for 28-joint counts based on the erythrocyte sedimentation rate (DAS28-4(ESR)) <2.6 response rates at: (1) month 9 (visit 5) of the blinded study (“-3 months” relative to the switch); (2) the time of switching from the blinded to the extension study (“switch”), and (3) month 3 (visit 4) of the extensions study (“+3 months”) allowing visit windows of ±1.5 months to capture all efficacy and safety data.

## Results

### Patient disposition and demographics

Of 204 patients randomized to ADA 40 mg Q2W + MTX in the blinded study, 107 switched to open-label tofacitinib 10 mg BID (105 (98 %) with MTX on day 1) within 2 weeks after their last dose of ADA. Of 201 patients randomized to blinded tofacitinib 10 mg BID + MTX in the blinded study, 126 switched to open-label tofacitinib 10 mg BID (123 (98 %) with MTX on day 1) within 2 weeks. Patient demographic and baseline characteristics were similar across sequences and between those patients who were randomized to ADA 40 mg Q2W + MTX or tofacitinib 10 mg BID + MTX in the blinded study and those who then received tofacitinib 10 mg BID (with or without MTX) in the extension study (Table 1).

**Table 1:** Baseline characteristics for patients randomized to double-blind adalimumab (ADA) 40 mg every 2 weeks or tofacitinib 10 mg twice daily (BID) in the phase 3 study [10] and for the subgroup who then received open-label tofacitinib 10 mg BID in the extension study [13]

	Double-blind study		Double-blind to open-label extension^a^	
	ADA 40 mg	Tofacitinib 10 mg	ADA to tofacitinib	Tofacitinib to tofacitinib
	n = 204	n = 201	n = 107	n = 126
Female, n (%)	162 (79.4)	168 (83.6)	83 (77.6)	107 (84.9)
White, n (%)	148 (72.5)	143 (71.1)	90 (84.1)	98 (77.8)
Age in years, mean ± SD	52.5 ± 11.7	52.9 ± 11.8	52.2 ± 11.4	51.6 ± 11.2
Region of origin, %				
North America	25.5	24.9	16.8	20.6
South America	2.9	1.5	4.7	1.6
Europe	53.9	55.7	60.7	59.5
Rest of world	17.6	17.9	17.8	18.3
Tender and swollen joints, mean				
Tender	26.7	26.1	28.0	25.6
Swollen	16.4	15.8	16.8	16.1
Mean HAQ-DI score	1.5	1.5	1.5	1.5
Mean DAS28-4(ESR)	6.4	6.5	6.4	6.4
Mean CRP (mg/L)	17.5	17.3	16.2	17.9
Positive for rheumatoid factor, %	68.2	66.2	66.4	65.9
Positive for anti-CCP, %	74.8	64.0	74.8	65.1
Prior therapy, n (%)				
TNF inhibitor	16 (7.8)	14 (7.0)	12 (11.2)	10 (7.9)
Non-TNF inhibitor biologic agent	3 (1.5)	4 (2.0)	1 (0.9)	3 (2.4)
Disease-modifying drug other than MTX	114 (55.9)	115 (57.2)	66 (61.7)	80 (63.5)
Concomitant therapy, n (%)				
Glucocorticoids	125 (61.3)	129 (64.2)	63 (58.9)	77 (61.1)
Lipid-lowering medication	10 (4.9)	10 (5.0)	6 (5.6)	6 (4.8)

^a^Baseline values at the start of the double-blind study; includes patients who completed treatment with ADA 40 mg every 2 weeks or tofacitinib 10 mg BID in the blinded study (or discontinued treatment for reasons other than a tofacitinib-related serious adverse event (AE)) and then enrolled in the extension study and switched treatment with minimal washout (≤2 weeks after their last dose of study drug in the blinded study). *CCP* cyclic citrullinated peptide, *CRP* C-reactive protein, *DAS28-4(ESR)* Disease Activity Score for 28-joint counts based on the erythrocyte sedimentation rate, *HAQ-DI* Health Assessment Questionnaire-Disability Index, *MTX* methotrexate, *RA* rheumatoid arthritis, *SD* standard deviation, *TNF* tumor necrosis factor

### Safety and tolerability

On analysis of the proportions of patients with TEAEs occurring 3 months prior to and 3 months after the switch, there was no clear pattern of changes in either the ADA to tofacitinib group or the blinded to open-label tofacitinib group (Table 2). Incidence rates per 100 patient-years of DCs due to AEs, serious AEs, and serious infections over 3-month and 12-month periods were similar in the year before and in the year after switching in both groups (Table 3). Incidence rates were increased in the first 3 months of the extension study (0 − 3 months) compared with the last 3 months of the blinded study (9 − 12 months) for both groups.

**Table 2:** Incidence of treatment-emergent adverse events (TEAEs), and the most commonly reported TEAEs (occurring in ≥2 % of patients in any group) during the last 3 months of the double-blind phase 3 study [10] and the first 3 months of the open-label extension [13]

	ADA to tofacitinib		Tofacitinib to tofacitinib	
	n = 107		n = 126	
	−3 months to switch (ADA)	Switch to +3 months (tofacitinib)	−3 months to switch (tofacitinib)	Switch to +3 months (tofacitinib)
Safety summary				
Number of patients (%)				
DCs due to AEs	0	3 (2.8)	1 (0.8)	3 (2.4)
Serious AEs	1 (0.9)	8 (7.5)	4 (3.2)	8 (6.3)
Serious infections	0	2 (1.9)	1 (0.8)	2 (1.6)
Deaths	0	2 (1.9)^a^	0	0
Most commonly reported TEAEs				
Number of patients (%)				
Abdominal pain upper	0	1 (0.9)	4 (3.2)	1 (0.8)
Arthralgia	0	1 (0.9)	4 (3.2)	2 (1.6)
Bronchitis	4 (3.7)	3 (2.8)	6 (4.8)	2 (1.6)
Cough	0	1 (0.9)	1 (0.8)	3 (2.4)
Depression	0	1 (0.9)	3 (2.4)	1 (0.8)
Fall	1 (0.9)	1 (0.9)	3 (2.4)	1 (0.8)
Hemoglobin decreased	0	3 (2.8)	0	1 (0.8)
Headache	0	2 (1.9)	0	2 (1.6)
Nasopharyngitis	2 (1.9)	4 (3.7)	5 (4.0)	3 (2.4)
Nausea	1 (0.9)	3 (2.8)	1 (0.8)	1 (0.8)
Edema peripheral	1 (0.9)	0	2 (1.6)	3 (2.4)
Oropharyngeal pain	0	0	0	3 (2.4)
Upper respiratory tract infection	3 (2.8)	5 (4.7)	3 (2.4)	6 (4.8)
Urinary tract infection	0	5 (4.7)	2 (1.6)	0
Vomiting	0	2 (1.9)	1 (0.8)	0
Worsening RA	0	0	3 (2.4)	2 (1.6)

Includes patients who completed treatment with adalimumab (ADA) 40 mg every 2 weeks or tofacitinib 10 mg twice daily in the blinded study (or discontinued treatment for reasons other than a tofacitinib-related serious adverse event (AE)) and then enrolled in the extension study and switched treatment with minimal washout (≤2 weeks after their last dose of study drug in the blinded study). ^a^One patient committed suicide 1 day after the first tofacitinib dose in the extension study (it is unknown whether the patient took the study drug on this day). One patient died due to lung malignancy. *DC* discontinuation, *RA* rheumatoid arthritis

**Table 3:** Adverse events and change from baseline in selected laboratory variables during the phase 3 double-blind study [10] and the open-label extension study [13]

	Double-blind study^a^					Open-label extension study^b^				
IR per 100 patient-years (95 % CI)	0 − 3 months	3 − 6 months	6 − 9 months	9 − 12 months	Total 12 months	0 − 3 months	3 − 6 months	6 − 9 months	9 − 12 months	Total 12 months
ADA 40 mg Q2W (blinded study) to tofacitinib 10 mg BID (extension study)										
	ADA 40 mg Q2W					Tofacitinib 10 mg BID				
	N = 204	N = 187	N = 177	N = 163	N = 204	N = 145	N = 130	N = 124	N = 121	N = 145
DCs due to AEs	16.3 (9.0, 29.5)	19.2 (9.6, 38.4)	6.7 (2.1, 20.7)	4.1 (0.6, 29.3)	12.9 (8.6, 19.4)	10.0 (4.2, 24.0)	9.6 (3.1, 29.9)	3.3 (0.5, 23.6)	12.0 (3.9, 37.2)	8.8 (5.0, 15.6)
Serious AEs	8.9 (4.0, 19.9)	16.8 (8.0, 35.2)	6.7 (2.2, 20.7)	8.2 (2.1, 32.9)	10.3 (6.5, 16.3)	16.1 (8.0, 32.2)	16.2 (6.8, 39.0)	6.7 (1.7, 26.7)	8.0 (2.0, 31.8)	13.0 (8.1, 20.9)
Serious infections	0	4.8 (1.2, 19.1)	2.2 (0.3, 15.8)	0	1.7 (0.5, 5.2)	4.0 (1.0, 15.9)	0	3.3 (0.5, 23.7)	4.0 (0.6, 28.1)	2.9 (1.1, 7.9)
Tofacitinib 10 mg BID (blinded study) to tofacitinib 10 mg BID (extension study)										
	Tofacitinib 10 mg BID					Tofacitinib 10 mg BID				
	N = 201	N = 184	N = 173	N = 157	N = 201	N = 146	N = 128	N = 122	N = 109	N = 146
DCs due to AEs	16.7 (9.3, 30.2)	22.2 (11.6, 42.7)	6.8 (2.2, 21.1)	0.0 (0.0, 0.0)	13.3 (8.8, 20.0)	13.9 (6.6, 29.1)	3.3 (0.5, 23.2)	10.5 (3.4, 32.5)	4.4 (0.6, 31.5)	9.1 (5.2, 16.0)
Serious AEs	20.1 (11.6, 34.5)	12.3 (5.1, 29.6)	9.1 (3.4, 24.2)	8.7 (2.2, 34.8)	13.7 (9.1, 20.6)	18.0 (9.4, 34.6)	9.9 (3.2, 30.6)	14.0 (5.3, 37.3)	4.4 (0.6, 31.5)	13.4 (8.3, 21.5)
Serious infections	4.6 (1.5, 14.2)	2.4 (0.3, 17.3)	4.5 (1.1, 18.1)	4.3 (0.6, 30.7)	4.0 (1.9, 8.4)	5.9 (1.9, 18.4)	3.3 (0.5, 23.2)	3.5 (0.5, 24.8)	4.4 (0.6, 31.5)	4.5 (2.0, 10.1)
	Double-blind study^b^					Long-term extension study^b^				
Mean change from baseline (SD)	Month 1	Month 3	Month 6	Month 9	Month 12	Month 1	Month 3	Month 6	Month 9	Month 12
ADA 40 mg Q2W (blinded study) to tofacitinib 10 mg BID (extension study)										
	ADA 40 mg Q2W					Tofacitinib 10 mg BID				
	N = 142	N = 142	N = 141	N = 138	N = 132	N = 132	N = 126	N = 118	N = 118	N = 110
Absolute lymphocyte count, cells/mm^3^	0.4 (0.6)	0.4 (0.6)	0.4 (0.6)	0.4 (0.6)	0.4 (0.7)	0.5 (0.8)	0.2 (0.6)	0.0 (0.6)	-0.0 (0.6)	-0.0 (0.6)
	N = 142	N = 144	N = 144	N = 140	N = 137	N = 139	N = 131	N = 125	N = 122	N = 115
Total cholesterol, mg/dL	8.3 (26.4)	6.7 (30.1)	2.8 (29.4)	-0.1 (29.7)	3.2 (33.9)	22.8 (33.2)	24.5 (38.2)	18.9 (36.4)	20.8 (35.6)	18.8 (37.7)
	N = 140	N = 141	N = 142	N = 139	N = 134	N = 1	N = 3	N = 121	N = 2	N = 107
LDL, mg/dL	3.2 (20.5)	2.5 (24.9)	0.5 (24.3)	-3.5 (23.1)	-1.8 (27.0)	18.9 (N/A)	-6.2 (57.8)	6.2 (30.2)	36.0 (25.5)	8.6 (31.9)
	N = 142	N = 144	N = 143	N = 140	N = 135	N = 1	N = 3	N = 122	N = 2	N = 108
HDL, mg/dL	3.3 (9.2)	3.4 (10.0)	1.6 (11.3)	3.3 (11.5)	5.6 (10.5)	20.9 (N/A)	13.1 (24.5)	11.9 (11.8)	10.5 (30.4)	8.8 (12.3)
	N = 142	N = 144	N = 143	N = 140	N = 135	N = 1	N = 5	N = 122	N = 3	N = 108
Triglycerides, mg/dL	7.8 (49.9)	2.8 (41.2)	2.7 (45.8)	-0.1 (58.7)	-6.0 (48.9)	5.3 (N/A)	2.0 (53.2)	4.7 (57.1)	8.0 (13.6)	3.1 (54.8)
Tofacitinib 10 mg BID (blinded study) to tofacitinib 10 mg BID (extension study)										
	Tofacitinib 10 mg BID					Tofacitinib 10 mg BID				
	N = 142	N = 141	N = 141	N = 138	N = 129	N = 134	N = 131	N = 116	N = 112	N = 103
Absolute lymphocyte count, cells/mm^3^	0.2 (0.5)	-0.0 (0.5)	-0.1 (0.6)	-0.1 (0.6)	-0.3(0.6)	-0.1 (0.6)	-0.2 (0.6)	-0.3 (0.6)	-0.3 (0.6)	-0.3 (0.6)
	N = 145	N = 142	N = 141	N = 136	N = 131	N = 136	N = 134	N = 122	N = 117	N = 106
Total cholesterol, mg/dL	28.7 (29.2)	27.4 (31.7)	30.0 (34.3)	29.3 (36.6)	32.8 (37.2)	30.6 (33.9)	27.6 (38.8)	22.5 (42.2)	23.7 (36.3)	29.7 (32.7)
	N = 141	N = 139	N = 137	N = 133	N = 123	N = 0	N = 5	N = 117	N = 4	N = 100
LDL, mg/dL	18.7 (25.6)	19.1 (25.5)	20.7 (31.0)	19.8 (31.1)	20.0 (31.8)	N/A	9.8(28.4)	13.1 (35.8)	16.3(42.5)	20.7 (30.7)
	N = 145	N = 142	N = 141	N = 136	N = 127	N = 0	N = 5	N = 121	N = 4	N = 103
HDL, mg/dL	8.0 (12.4)	6.1 (14.1)	7.7 (17.9)	7.1 (15.4)	8.2 (15.7)	N/A	16.1 (19.0)	8.6 (19.1)	7.9 (3.9)	9.0 (15.2)
	N = 145	N = 142	N = 141	N = 136	N = 127	N = 0	N = 7	N = 121	N = 4	N = 103
Triglycerides, mg/dL	9.2 (65.0)	8.4 (69.4)	13.0 (87.3)	12.3 (91.0)	15.0 (83.3)	N/A	25.5 (69.2)	12.9 (84.8)	6.6 (61.7)	7.5 (70.1)

^**a**^Includes patients who were treated with adalimumab (ADA) 40 mg every 2 weeks (Q2W) or tofacitinib 10 mg twice daily (BID) from baseline in the blinded study, regardless of whether they entered the extension study. ^**b**^Includes all patients who completed treatment with ADA 40 mg Q2W or tofacitinib 10 mg BID in the blinded study and then switched treatment and completed 12 months of the extension study. Please see “Methods” for important exceptions. *AE* adverse event, *CI* confidence interval, *DC* discontinuation, *HDL* high-density lipoprotein, *IR* incidence rate, *LDL* low density lipoprotein, *N/A* not available

In the extension study, small increases from baseline in ALC were observed at month 1 and month 3 in patients in the ADA to tofacitinib group; ALC levels returned to baseline at month 6 and were maintained through month 12. Small decreases from baseline in ALC were observed at all time points through month 12 in patients in the blinded to open-label tofacitinib group (Table 3). In the extension study, patients in the ADA to tofacitinib group and patients in the blinded to open-label tofacitinib group had small increases from baseline in total cholesterol, low-density lipoprotein (LDL), high-density lipoprotein (HDL) and triglycerides at all time points through month 12 (except for LDL at month 3 in patients in the ADA to tofacitinib group) (Table 3). At month 3 of the extension study, 2 patients (2 %) in the ADA to tofacitinib group and 4 patients (3 %) in the blinded to open-label tofacitinib group were receiving lipid-lowering agents.

Serious AEs occurring up to the month-3 visit of the extension study - that is, following the switch to tofacitinib - are presented in Table 4 alongside the action taken and patient outcomes. Two serious AEs resulted in death (lung malignancy, n = 1 (day 132); suicide, n = 1 (day 1); it is unknown whether the patient took the study drug on this day). One patient who developed vertigo during this period was still experiencing it 20 months later. All other patients who experienced a serious AE in the first 3 months of the extensions study recovered from the AE.

**Table 4:** Patient-level serious adverse events from switch to +3 months

Serious AE	Days after first tofacitinib dose in the extension study	Action taken (tofacitinib dosing)	Outcome
ADA 40 mg Q2W (blinded study) to tofacitinib 10 mg BID (extension study), N = 107			
Suicide (n = 1)	1^a^	Permanently withdrawn	Death
Osteonecrosis (n = 1)	23	Temporarily withdrawn	Recovered
Post-operative abscess (n = 1)	44	Dose not changed	Recovered
Infectious peritonitis/appendicitis/intervertebral disc disorder (n = 1 each; same patient)	44/46/51	Permanently withdrawn	Recovered
Autoimmune hepatitis (possible drug-induced liver injury; n = 1)	55	Permanently withdrawn	Recovered
Fractured ischium (n = 1)	109	Temporarily withdrawn	Recovering
Spondylolisthesis/stenosis/lung malignancy (n = 1 each; same patient)	117/117/132	Not applicable	Death^b^
Fractured femur (n = 1)	134	Temporarily withdrawn	Recovered
Tofacitinib 10 mg BID (blinded study) to tofacitinib 10 mg BID (extension study), N = 126			
Sinusitis (n = 1)	13	Permanently withdrawn	Recovered
Dyspnea/tendon rupture (n = 1 each; same patient)	37/53	Temporarily withdrawn	Recovered
Cervical dysplasia (n = 1)	52	Permanently withdrawn	Recovered
Malignant melanoma (n = 1)	85	Permanently withdrawn	Recovered
Vertigo (n = 1)	89	Dose not changed	Not recovered
Disseminated herpes simplex (n = 1)	94	Permanently withdrawn	Recovered
Cervical vertebral fracture/fall (n = 1 each; same patient)	102/102	Dose not changed	Recovering
Lung infiltration/atrial fibrillation (n = 1 each; same patient)^c^	107/122	Permanently withdrawn	Recovered/not applicable
Goiter (n = 1)	120	Temporarily withdrawn	Recovered

Includes patients who completed treatment with adalimumab (ADA) 40 mg every 2 weeks (Q2W) or tofacitinib 10 mg twice daily (BID) in the blinded study (or discontinued treatment for reasons other than a tofacitinib-related serious adverse event (AE)) and then enrolled in the extension study and switched treatment with minimal washout (≤2 weeks after their last dose of study drug in the blinded study). ^a^Unknown whether the patient took study drug on this day. ^b^Due to lung malignancy. ^c^Atrial fibrillation (day 122) occurred after drug discontinuation (day 107)

### Efficacy

**Fig. 1:**
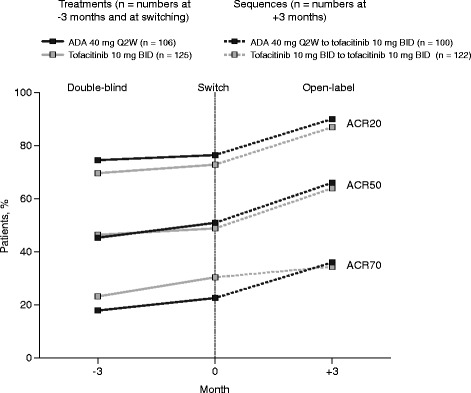
American College of Rheumatology (*ACR*) response rates 3 months before switch, at switch, and 3 months after switch*. *ADA* adalimumab, *BID* twice daily. *Includes patients who completed treatment with ADA 40 mg Q2W or tofacitinib 10 mg BID in ORAL Standard (or discontinued treatment for reasons other than a tofacitinib-related serious AE) and then enrolled in ORAL Sequel and switched treatment with minimal washout (≤2 weeks after their last dose of study drug in ORAL Standard). ACR, American College of Rheumatology; ACR20/50/70, 20/50/70% reduction in the number of tender and swollen joints, as well as 20/50/70% improvement in ≥3 of the other five ACR components; ADA, adalimumab; BID, twice daily; Q2W, every 2 weeks

**Fig. 2:**
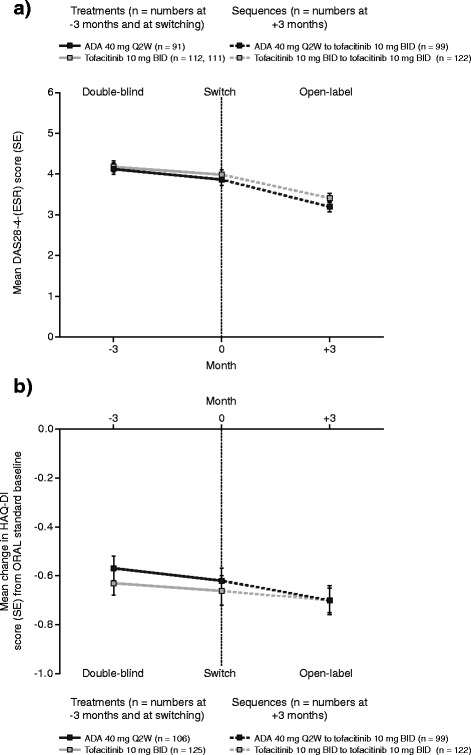
Disease Activity Score for 28-joint counts based on the erythrocyte sedimentation rate (*DAS28-4(ESR*)) and changes from baseline in the Health Assessment Questionnaire-Disability Index (*HAQ-DI*) 3 months before switch, at switch, and 3 months after switch*. *ADA* adalimumab, *BID* twice daily. *Includes patients who completed treatment with ADA 40 mg Q2W or tofacitinib 10 mg BID in ORAL Standard (or discontinued treatment for reasons other than a tofacitinib-related serious AE) and then enrolled in ORAL Sequel and switched treatment with minimal washout (≤2 weeks after their last dose of study drug in ORAL Standard). Lower scores indicate less disease activity (DAS28–4[ESR]) or lower disability (HAQ-DI). ADA, adalimumab; BID, twice daily; DAS28–4(ESR), Disease Activity Score for 28 joint counts based on the erythrocyte sedimentation rate; HAQ-DI, Health Assessment Questionnaire-Disability Index; Q2W, every 2 weeks; SE, standard error. (**a**) Changes from Baseline in Disease Activity Score for 28-joint counts based on Erythrocyte sedimentation rate (DAS28-4(ESR) 3 months before switch, at switch, and 3 months after switch.* (**b**) Changes from Baseline in the Health Assessment QUestionnaire-Disability Index (HAQ-DI) 3 months before switch, at switch, and 3 months after switch*

Greater proportions of patients had improvement in the signs and symptoms of RA, as measured by increased ACR response rates (Fig. 1) and reductions in mean DAS28-4(ESR), during the first 3 months of the extension study compared with the last 3 months of the blinded study in both the ADA to tofacitinib group and the blinded to open-label tofacitinib group (Fig. 2a). Of the 74 patients on ADA and 84 patients on tofacitinib who achieved an ACR20 response 3 months prior to switching in the double-blind study, 7 % (n = 5) and 7 % (n = 6), respectively, did not maintain an ACR20 response 3 months after switching to tofacitinib in the extension study.

Similarly, further improvement in physical function, as measured by greater mean reductions in HAQ-DI score from baseline, was seen in both treatment groups (Fig. 2b).

## Discussion

The clinical, functional, and radiographic efficacy and safety of tofacitinib 5 and 10 mg BID with and without MTX has been reported in patients with active RA in randomized phase 2 [15–19] and phase 3 studies [7–12]. The phase 3 study that included an ADA treatment arm demonstrated efficacy and safety results of tofacitinib consistent with the other phase 3 studies [10]. The current analyses describe the safety and efficacy of open-label tofacitinib (with or without MTX) following a direct switch from blinded treatment with either ADA + MTX or tofacitinib + MTX.

Both proportions and exposure-adjusted incidence rates of DCs due to AEs, serious AEs, and serious infections over time were similar when switching from blinded ADA or tofacitinib to open-label tofacitinib. The increased incidence rates for these events in the initial 3 months of open-label tofacitinib, compared with the last 3 months of either blinded ADA or tofacitinib, suggests that these differences may be due to aspects of the study design inherent in the transition period rather than as a result of any overlapping immunomodulatory effects of ADA and tofacitinib. One potential explanation for the observed changes is the possibility of a measurement/detection effect. For example, the transition from the double-blind study to the open-label extension study may have driven an increase in patient/investigator sensitivity to reporting, whereby there was increased attribution of events in the extension study. Another limitation could have been the number of visits per 3-month period. Patients in the extension study had more frequent visits over the first 3 months of treatment (four visits) than they had in the final 3 months of the blinded study (two visits); thus, there were more opportunities to report an event in the first 3 months after the switch, which could have had an impact on the apparent incidence rate. Although a mechanistic or immunologic origin for the difference in safety profile cannot be ruled out, it is thought to be unlikely, as an increase was observed in both treatment sequences. On analysis of the proportions of patients experiencing TEAEs for 3 months before and 3 months after the switch, there was no clear pattern of changes during the switch. A further limitation is the potential change in dosing of permitted concomitant RA medications, including MTX, after switching.

Although the analyses of incidence rates per 100 patient-years of AEs in the blinded study included all patients regardless of whether they entered the extension, the data for the extension study included only those patients who completed the blinded study (that is, those who had data for the last 3 months of this study and who then enrolled in the extension). This introduces a potential censoring/truncation effect that is associated with identifying and analyzing a “completer” population. This effect may also apply to the efficacy response: patients with lesser efficacy responses may have been less likely to remain in the extension study to month 3, thereby, leading to seemingly greater improvements at month 3 among patients in this study. However, the incidence of both DCs and improvements in efficacy during the first 3 months of the extension study were similar between those patients who switched to ADA and those who continued to receive tofacitinib.

With these limitations in mind, we observed that switching directly from double-blind ADA 40 mg Q2W to open-label tofacitinib 10 mg BID resulted in sustained clinical response, with numeric improvements in all ACR response rates assessed, DAS28-4(ESR) scores, and mean change from baseline in HAQ-DI scores. Efficacy assessed at the same time points was similar in the double-blind tofacitinib 10 mg BID to open-label tofacitinib 10 mg BID group and the ADA to tofacitinib 10 mg BID group, and there was a similar pattern of increases in efficacy from the double-blind to the open-label extension.

One of the questions of interest was whether there are clinical consequences of potential overlapping immunomodulatory effects in the switch from ADA to tofacitinib. Analysis of AEs do not support such a conclusion but, in contrast, the contribution that overlapping pharmacologic exposure to ADA and tofacitinib makes to efficacy may be more difficult to assess. Although the first efficacy assessment at 3 months is well beyond the terminal half-life of the last dose of ADA [20, 21], pharmacodynamic effects may persist beyond what might be predicted from half-life/clearance data, and co-administered MTX reduces ADA clearance [22]. Nevertheless, efficacy assessed at the same time points was similar in the tofacitinib to tofacitinib group and the ADA to tofacitinib group, with similar numeric patterns of improvement.

## Conclusions

In summary, analysis of incidence rates of AEs showed a similar pattern over time for both ADA to tofacitinib and blinded to open-label tofacitinib treatment sequences in the year before and the year after switching, and there was no clear pattern of changes in TEAEs in the 3 months before and 3 months after the switch. Switching from both double-blind ADA and tofacitinib to open-label tofacitinib resulted in numeric improvements in signs and symptoms of disease and physical function. These results indicate that a patient’s treatment can be directly switched from ADA to tofacitinib 10 mg BID, with a safety and efficacy profile similar to switching from blinded to open-label tofacitinib 10 mg BID. This analysis did not address potential differences between the safety profiles of ADA and tofacitinib in the phase 3 study for this cohort of patients, as this comparison is more appropriate in the primary safety analysis population [10].

## Abbreviations

ACR, American College of Rheumatology; ADA, adalimumab; AE, adverse event; ALC, absolute lymphocyte count; BID, twice daily; CCP, cyclic citrullinated peptide; CI, confidence interval; CRP, C-reactive protein; DAS28-4(ESR), Disease Activity Score for 28-joint counts based on the erythrocyte sedimentation rate; DC, discontinuation; DMARD, disease-modifying anti-rheumatic drug; HAQ-DI, Health Assessment Questionnaire-Disability Index; IR, incidence rate; LDL, high-density lipoprotein; MTX, methotrexate; Q2W, every 2 weeks; RA, rheumatoid arthritis; HDL, high-density lipoprotein; SD, standard deviation; TEAE, treatment-emergent adverse event; TNFi, tumor necrosis factor inhibitor

## Additional file

Additional file 1: Table S1. Ethical review boards and study centers. (DOCX 34 kb)
