# Supplementary material for: Switching from adalimumab to tofacitinib in the treatment of patients with rheumatoid arthritis
Source: Arthritis Res Ther. 2016 Jun 23;18:145. doi: 10.1186/s13075-016-1049-3 (PMC4918072; doi:10.1186/s13075-016-1049-3)
Supplement: Additional file 1: Table S1. — Ethical review boards and study centers. (DOCX 34 kb) [file 13075_2016_1049_MOESM1_ESM.docx]

**Switching from adalimumab to tofacitinib in the treatment of patients with rheumatoid arthritis**

Mark C. Genovese, Ronald van Vollenhoven, Bethanie Wilkinson, Lisy Wang, Samuel H. Zwillich, David Gruben, Pinaki Biswas, Richard Riese, Liza Takiya, Thomas V. Jones

**Additional file 1: Table S1.** Ethical review boards and study centers

| **Study Center** | **Institutional Review Board or Ethics Committee** |
| --- | --- |
| Consulta Privada Dra. Marta Aliste  Guardia Vieja 255, Oficina 1409  Providencia  Santiago, RM 7510186  CHILE | Comite Etico Cientifico, Servicio de  Salud Metropolitano Oriente  Avenida Salvador 364  Providencia  Santiago, RM 7500922  CHILE |
| Centro de Diagnostico y  Tratamiento San Borja Arriaran  Seccion Reumatologia  Amazonas 619  Santiago, RM 8360156  CHILE | Comite Etico Cientifico  Servicio de Salud Metropolitano  Oriente  Avenida Salvador 364  Providencia, Santiago, RM 7500922  CHILE |
| Latin American Research  Avenida Maximo Gomez #60,  suite 201, plaza paseo del treatro  Santo Domingo, Santo Domingo  00000  DOMINICAN REPUBLIC | CONABIOS  Universidad Católica Santo Domingo  Calle Santo Domingo No3, Ens. La  Julia  Santo Domingo, Santo Domingo  2733  DOMINICAN REPUBLIC |
| MBAL na Voennomeditsinska  Akademia - Sofia  Klinika po Revmatologia i  Kardiologia  MMA HAT Sofia  Ul. "Georgi Sofiyski" 3  Sofia, 1606  BULGARIA | Ethics Committee for Multicenter  Trials  ul. "Damyan Gruev" 8  Sofia, 1303  BULGARIA  Komisiya po etika pri MBAL na  Voennomeditsinska Akademia -  Sofia/Ethics Commettee at MMA  HAT-Sofia  MMA HAT-Sofia  Ul. Georgi Sofiyski  3  Sofia, 1606  BULGARIA |
| UMBAL "D-r Georgi Stranski"  Pleven  Klinika po Kardiologia i  Revmatologia  MHAT "Dr. Georgi Stranski"  Pleven  Ul. "Georgi Kochev" 8A  Pleven, 5800  BULGARIA | Ethics Committee for Multicenter  Trials  ul. "Damyan Gruev" 8  Sofia, 1303  BULGARIA  Komisiya po etika pri UMBAL"D-r  Georgi Stranski"/ Ethics Committee  at MHAT "Dr. Georgi Stranski"  UMBAL"D-r Georgi Stranski"  Pleven  MHAT "Dr. Georgi Stranski" Pleven  Ul. Georgi Kochev  8A  Pleven, 5800  BULGARIA |
| MBAL "Kaspela" Plovdiv,  Otdelenie po revmatologia  MHAT "Kaspela" Plovdiv  ul. Sofiya  64  Plovdiv, 4002  BULGARIA | Ethics Committee for Multicenter  Trials  ul. "Damyan Gruev" 8  Sofia, 1303  BULGARIA  Komisiya po etika kam MBAL  "Kaspela"/ Ethics committee at  MHAT "Kaspela"  MBAL 'Kaspela' Plovdiv  MHAT 'Kaspela' Plovdiv  Ul. Sofiya 64  Plovdiv, 4002  BULGARIA |
| The Arthritis Research Centre of  Canada  895 W 10th Avenue  Vancouver, BC V5Z 1L7  CANADA | IRB Services  Suite 300  372 Hollandview Trail  Aurora, ON L4G 0A5  CANADA |
| Centre de rhumatologie St-Louis  Bureau 140  3165 chemin St-Louis  Quebec, QC G1W 4R4  CANADA | IRB Services  Suite 300  372 Hollandview Trail  Aurora, ON L4G 0A5  CANADA |
| Centre de Recherche Musculo-  Squelettique  1119 Ste Marguerite  Trois-Rivieres, QC G8Z 1Y2  CANADA | IRB Services  Suite 300  372 Hollandview Trail  Aurora, ON L4G 0A5  CANADA |
| Royal University Hospital  Room 367 Ellis Hall  103 Hospital Drive  Saskatoon, SK S7N 0W8  CANADA | University of Saskatchewan  Biomedical Research Ethics Board  Ethics Office  P.O. Box 5000 RPO University  Saskatoon, SK S7N 0W8  CANADA  University of Saskatchewan  BioMedical Research Ethics Board  (Bio-REB)  Research Ethics Office, University of  Saskatchewan  NRC, Plant Biotechnology Research  Institute  1607 110 Gymnasium Place  Saskatoon, SK S7N 4J8  CANADA |
| NEA Baptist Clinic  311 East Matthews Avenue  Jonesboro, AR 72401  UNITED STATES  NEA Baptist Clinic  Clinical Research Center  Suite C  416 East Washington Avenue  Jonesboro, AR 72401  UNITED STATES | Quorum Institutional Review Board  Suite 1000  1601 Fifth Avenue  Seattle, WA 98101  UNITED STATES |
| Kiljavan Laaketutkimus Oy  Donnerinkatu 5  Hyvinkaa, 05800  FINLAND | Helsingin ja Uudenmaan  sairaanhoitopiiri  Sisatautien eettinen toimikunta  Biomedicum Helsinki 2 C  PL 705  HUS, 00029  FINLAND |
| HOSPITAL NUESTRA  SEÑORA DE LA ESPERANZA  AVENIDA DE LAS BURGAS, 2  SANTIAGO DE  COMPOSTELA, A CORUÑA  15705  SPAIN | Comité Ético de Investigación de  Galicia (SERGAS)  ETHICS COMMITTEE OF CLINIC  INVESTIGATION  DIVISION DE FARMACIA Y  PRODUCTOS  C/ SAN LAZARO, S/N  CONSELLERIA DE SANIDADE  SANTIAGO DE COMPOSTELA, A  CORUÑA 15703  SPAIN |
| COMPLEJO HOSPITALARIO  UNIVERSITARIO A CORUÑA  LABORATORIO DE  INVESTIGACION. EDIFICIO  ANEXO AL HOSPITAL  MATERNO INFANTIL  C/. XUBIAS DE ARRIBA, 84  A CORUÑA, A CORUÑA 15006  SPAIN | Comité Ético de Investigación de  Galicia (SERGAS)  ETHICS COMMITTEE OF CLINIC  INVESTIGATION  DIVISION DE FARMACIA Y  PRODUCTOS  C/ SAN LAZARO, S/N  CONSELLERIA DE SANIDADE  SANTIAGO DE COMPOSTELA, A  CORUÑA 15703  SPAIN |
| Med Investigations  (Administrative Office)  Suite 260  6600 Mercy Court  Fair Oaks, CA 95628  UNITED STATES  Office of Douglas M. Haselwood,  MD  Suite 1201  151 North Sunrise Avenue  Roseville, CA 95661  UNITED STATES | Quorum Institutional Review Board  Suite 1000  1601 Fifth Avenue  Seattle, WA 98101  UNITED STATES |
| Tampa Medical Group, P.A.  Suite 406  13801 Bruce B. Downs  Boulevard  Tampa, FL 33613  UNITED STATES | Quorum Institutional Review Board  Suite 1000  1601 Fifth Avenue  Seattle, WA 98101  UNITED STATES |
| Austin Rheumatology Research  Suite 702  1301 West 38th Street  Austin, TX 78705  UNITED STATES  Austin Rheumatology Research  Suite 110  1301 West 38th Street  Austin, TX 78705  UNITED STATES | Quorum Institutional Review Board  Suite 1000  1601 Fifth Avenue  Seattle, WA 98101  UNITED STATES |
| Bluegrass Community Research,  Inc.  330 Waller Avenue  Lexington, KY 40504  UNITED STATES | Quorum Institutional Review Board  Suite 1000  1601 Fifth Avenue  Seattle, WA 98101  UNITED STATES |
| Ochsner Clinic Foundation,  Baton Rouge  2nd Floor Research  9001 Summa Avenue  Baton Rouge, LA 70809  UNITED STATES | Ochsner Clinic Foundation  Institutional Review Board  1514 Jefferson Highway  New Orleans, LA 70121  UNITED STATES |
| Seoul National University  Hospital, Rheumatology, Internal  Medicine  28 Yongon-dong, Chongno-gu  Seoul, 110-744  KOREA, REPUBLIC OF | Seoul National University Hospital  Institutional Review Board  28 Yongon-dong, Chongno-gu  Seoul, 110-744  KOREA, REPUBLIC OF |
| Hanyang University Hospital,  Department of Rheumatology  17 Hengdang-dong, Seongdonggu  Seoul, 133-792  KOREA, REPUBLIC OF | Hanyang University Hospital IRB  17 Haengdang-dong, Seondong-gu  Seoul, 133-792  KOREA, REPUBLIC OF |
| Chonnam National University  Hospital  8, Hak-dong, Dong-gu  Gwangju, 501-757  KOREA, REPUBLIC OF | IRB of Chonnam National University  Hospital  8, Hak-dong, Dong-gu  Gwangju, 501-757  KOREA, REPUBLIC OF |
| Marietta Rheumatology  Suite 100  670 North Avenue  Marietta, GA 30060  UNITED STATES | Quorum Institutional Review Board  Suite 1000  1601 Fifth Avenue  Seattle, WA 98101  UNITED STATES |
| Krakowskie Centrum Medyczne  NZOZ  Kopernika 32  Krakow, 31-501  POLAND | Komisja Bioetyczna przy Okregowej  Izbie Lekarskiej w Gdansku  ul. Sniadeckich 33  Gdansk, 80-204  POLAND |
| Wojewodzki Zespol  Reumatologiczny im. dr J. Titz-  Kosko  ul. Grunwaldzka 1/3  Sopot, 81-759  POLAND | Komisja Bioetyczna przy Okregowej  Izbie Lekarskiej w Gdansku  ul. Sniadeckich 33  Gdansk, 80-204  POLAND |
| Mountain State Clinical Research  Suite 303A  300 Davison Road  Clarksburg, WV 26301  UNITED STATES | Quorum Institutional Review Board  Suite 1000  1601 Fifth Avenue  Seattle, WA 98101  UNITED STATES |
| Hospital Universitari i Politecnic  La Fe de Valencia  Servicio de Reumatologia. Torre  B. 6ª Planta.  Bulevar Sur, s/n  Valencia, Valencia 46026  SPAIN | Comité Ético de Investigación de  Galicia (SERGAS)  ETHICS COMMITTEE OF CLINIC  INVESTIGATION  DIVISION DE FARMACIA Y  PRODUCTOS  C/ SAN LAZARO, S/N  CONSELLERIA DE SANIDADE  SANTIAGO DE COMPOSTELA, A  CORUÑA 15703  SPAIN |
| Cannock Rheumatology Dept.,  Cannock Chase Hospital  Brunswick Road  Cannock, Staffs WS11 2XY  UNITED KINGDOM | West Midlands Research Ethics  Commitee  West Midlands Research Ethics  Commitee  Prospect House  Fishing Line Road  Enfield, Redditch B97 6EW  UNITED KINGDOM |
| Rheumatology Research  5th Floor Block B  Cairns Base Hospital  The Esplanade  Cairns, QLD 4870  AUSTRALIA | Cairns and Hinterland Health Service  District Human Research Ethics  Committee  4th Floor, Block A  Cairns Base Hospital  Cairns, QLD 4870  AUSTRALIA |
| Emeritus Research  291 Wattletree Road  Malvern East, VIC 3145  AUSTRALIA | Cabrini Human Research Ethics  Committee  183 Wattletree Road  Malvern, VIC 3144  AUSTRALIA |
| Rheumatology Research Unit  Sunshine Coast  9-10 Maroochy Waters Shopping  Centre  Denna Street  Maroochydore, QLD 4558  AUSTRALIA | Redcliffe-Caboolture Ethics  Committee  Unit 1, Ground Floor, Redcliffe  Hospital  Anzac Avenue  Redcliffe, Qld 4020  AUSTRALIA |
| DKTs "Sveta Anna", Sofia;  Konsultativen kabinet po  Revmatologia  Diagnostic Consultative Center  "Sveta Anna"  Ul. Dimitar Mollov 1  Sofia, 1709  BULGARIA | Ethics Committee for Multicenter  Trials  ul. "Damyan Gruev" 8  Sofia, 1303  BULGARIA  Komisiya po etika pri DKTs"Sv.  Anna"/ Ethics Committee at DCC  "Sv. Anna"  DKTs "Sveta Anna"  Diagnostic Consultative Center  "Sveta Anna"  Ul. Dimitar Mollov 1  Sofia, 1709  BULGARIA |
| HOSPITAL 12 DE OCTUBRE  SERVICIO DE  REUMATOLOGIA - PLANTA  SEMISOTANO  AVDA. DE CORDOBA S/N  MADRID, MADRID 28041  SPAIN | Comité Ético de Investigación de  Galicia (SERGAS)  ETHICS COMMITTEE OF CLINIC  INVESTIGATION  DIVISION DE FARMACIA Y  PRODUCTOS  C/ SAN LAZARO, S/N  CONSELLERIA DE SANIDADE  SANTIAGO DE COMPOSTELA, A  CORUÑA 15703  SPAIN |
| Schlosspark-Klinik, Innere  Medizin II, Rheumatologie  Heubnerweg 2  Berlin, 14059  GERMANY | Ethik-Kommission des Fachbereichs  Medizin der Justus-Liebig-  Universitaet Giessen  Gaffkystrasse 11 c  Giessen, 35385  GERMANY |
| Unidad de Investigacion en  Enfermedades Cronico  Degenerativas  Colomos 2292, Providencia  Guadalajara, Jalisco 44620  MEXICO | Comite de Bioetica de la Unidad de  Investigacion en Enfermedades  Cronico-Degenerativas  Colomos 2292  Col. Providencia  Guadalajara, Jalisco 44620  MEXICO |
| Evangelisches Fachkrankenhaus  Ratingen gGmbH  Rosenstr. 2  Ratingen, 40882  GERMANY | Ethik-Kommission des Fachbereichs  Medizin der Justus-Liebig-  Universitaet Giessen  Gaffkystrasse 11 c  Giessen, 35385  GERMANY |
| Mary Mediatrix Medical Center  3rd Floor HB Calleja Bldg.  J.P. Laurel Highway  Lipa City, Batangas 4217  PHILIPPINES | Mary Mediatrix Medical Center  Institutional Review Board  Mary Mediatrix Medical Center  J.P. Laurel Highway  Lipa City, Batangas  PHILIPPINES |
| NZOZ CENTRUM MEDYCZNE  ul. Pulaskiego 69  Bialystok, 15-337  POLAND | Komisja Bioetyczna przy Okregowej  Izbie Lekarskiej w Gdansku  ul. Sniadeckich 33  Gdansk, 80-204  POLAND |
| DC Mediscan  Sustova 1930  Praha 11 - Chodov, 148 00  CZECH REPUBLIC  MUDr. Dana Sinaglova  Soukroma laborator klinicke  biochemie a hematologie  Sustova 1930  Praha 11, 14800  CZECH REPUBLIC  Sdruzene zdravotnicke zarizeni  JM II  Radiodiagnosticke oddeleni  Sustova 1930  Praha 11, 14800  CZECH REPUBLIC  Sdruzene zdravotnicke zarizeni  JM II  Lekarna  Sustova 1930  Praha 11, 14800  CZECH REPUBLIC | Eticka komise IKEM a FTNsP  Videnska 800  Praha 4 Krc, 140 59  CZECH REPUBLIC |
| Fakultni nemocnice u sv. Anny v  Brne  II. Interni klinika  Pekarska 53  Brno, 656 91  CZECH REPUBLIC | Eticka komise Fakultni nemocnice u  sv. Anny v Brne  Pekarska 53  Brno, 656 91  CZECH REPUBLIC  Eticka komise IKEM a FTNsP  Videnska 800  Praha 4 Krc, 140 59  CZECH REPUBLIC |
| PV-Medical s.r.o.  Revmatologicka ambulance  Padelky I/3645  Zlin, 760 01  CZECH REPUBLIC | Eticka komise IKEM a FTNsP  Videnska 800  Praha 4 Krc, 140 59  CZECH REPUBLIC |
| Revmatologicky ustav  Na Slupi 4  Praha 2, 128 50  CZECH REPUBLIC | Eticka komise IKEM a FTNsP  Videnska 800  Praha 4 Krc, 140 59  CZECH REPUBLIC |
| ARTHROMED, s. r. o.  Revmatologicka ambulance  Rokycanova 2798  Pardubice, 530 02  CZECH REPUBLIC | Eticka komise IKEM a FTNsP  Videnska 800  Praha 4 Krc, 140 59  CZECH REPUBLIC |
| Angeles University Foundation  Medical Center  3rd Floor Infection Control and  Research Room  Angeles City, Pampanga 2009  PHILIPPINES | Institutional Review Board  College of Medicine  3rd Floor San Agustin Building  Angeles University Foundation  Medical Center  McArthur Highway  Angeles City, Pampanga 2009  PHILIPPINES |
| Division of Rheumatology,  Department of Internal Medicine,  Faculty of Medicine  Chiang Mai University  110 Intavaroros Road  Amphoe Muang, Chiang Mai  50200  THAILAND | Research Ethics Committee 1  Research Ethics Committee 1,  Faculty of Medicine, Chiang Mai  University  110 Intavaroros Road  Amphoe Muang, Chiang Mai 50200  THAILAND |
| Cincinnati Rheumatic Disease  Study Group, Inc.  at the Deaconess Arthritis Center  311 Straight Street  Cincinnati, OH 45219  UNITED STATES | Quorum Institutional Review Board  Suite 1000  1601 Fifth Avenue  Seattle, WA 98101  UNITED STATES |
| San Diego Arthritis Medical  Clinic  Suite 300  3633 Camino Del Rio South  San Diego, CA 92108  UNITED STATES | Quorum Institutional Review Board  Suite 1000  1601 Fifth Avenue  Seattle, WA 9810 |
| Metroplex Clinical Research  Center  Suite 810  8144 Walnut Hill Lane  Dallas, TX 75231  UNITED STATES | Quorum Institutional Review Board  Suite 1000  1601 Fifth Avenue  Seattle, WA 98101  UNITED STATES |
| Nestatna reumatologicka  ambulancia, MUDr. Pavol Polak,  s.r.o.  Vojtecha Spanyola 43  Zilina, 010 01  SLOVAKIA | Eticka komisia  Zilinskeho samospravneho kraja  Komenskeho 48  Zilina, 011 09  SLOVAKIA  Eticka komisia, Trenciansky  samospravny kraj  K dolnej stanici 7282/20A  Trencin, 911 01  SLOVAKIA |
| Reumatologicka ambulancia,  Ecclesia, s.r.o.  SNP 42/A  Nove Zamky, 94001  SLOVAKIA | Eticka komisia Nitrianskeho  samospravneho kraja  Stefanikova trieda 69  Nitra, 949 60  SLOVAKIA  Eticka komisia, Trenciansky  samospravny kraj  K dolnej stanici 7282/20A  Trencin, 911 01  SLOVAKIA |
| AAGS, s.r.o. , nestatne  zdravotnicke zariadenie  Reumatologicka ambulancia  Velkoblahovska 10  Dunajska Streda, 92901  SLOVAKIA | Eticka komisia  Trnavskeho samospravneho kraja  Starohajska 10  Trnava, 917 01  SLOVAKIA  Eticka komisia, Trenciansky  samospravny kraj  K dolnej stanici 7282/20A  Trencin, 911 01  SLOVAKIA |
| Department of Rheumatology  Dudley Group of Hospitals NHS  Trust  Esk House, Russells Hall  Hospital  Dudley, West Midlands, DY1  2HQ  UNITED KINGDOM | West Midlands Research Ethics  Commitee  West Midlands Research Ethics  Commitee  Prospect House  Fishing Line Road  Enfield, Redditch B97 6EW  UNITED KINGDOM |
| BayCare Outpatient Imaging at  Bardmoor  (X-Ray Only)  8787 Bryan Dairy Road  Largo, FL 33777  UNITED STATES  DMI Research, Inc.  6699 90th Avenue North  Pinellas Park, FL 33782  UNITED STATES  St. Petersburg Arthritis Center  6711 38th Avenue North  St. Petersburg, FL 33710  UNITED STATES | Quorum Institutional Review Board  Suite 1000  1601 Fifth Avenue  Seattle, WA 98101  UNITED STATES |
| Revmatologichno Otdelenie,  MBAL - Plovdiv  MHAT Plovdiv  bul. Bulgaria 234  Plovdiv, 4000  BULGARIA | Ethics Committee for Multicenter  Trials  ul. "Damyan Gruev" 8  Sofia, 1303  BULGARIA  Komisiya po etika pri MBALPlovdiv/  Ethics Committee at MHATPlovdiv  MBAL-Plovdiv  MHAT-Plovdiv  bul. ¿Bulgaria¿ 234  Plovdiv, 4000  BULGARIA |
| ArthroCare, Arthritis Care &  Research P.C.  Suite 200  3921 East Baseline Road  Gilbert, AZ 85234  UNITED STATES | Quorum Institutional Review Board  Suite 1000  1601 Fifth Avenue  Seattle, WA 98101  UNITED STATES |
| Privat-Praxis, Rheumatologie  (P515)  Karlsgraben 15  Aachen, 52064  GERMANY | Ethik-Kommission des Fachbereichs  Medizin der Justus-Liebig-  Universitaet Giessen  Gaffkystrasse 11 c  Giessen, 35385  GERMANY |
| Rheumatology Unit, Department  of Internal Medicine,  Phramongkutklao Hospital  315  Rajavithi Road,  Rajathevee, Bangkok 10400  THAILAND | Institutional Review Board Royal  Thai Army Medical Department  317 Rajavithi Road  Rajathevee  Bangkok, 10400  THAILAND |
| University Hospital Center  Zagreb  Kispaticeva 12  Zagreb, 10000  CROATIA | Central Ethics Committee  Agency for Medicinal Products and  Medical Devices  Ksaverska c. 4  Zagreb, 10000  CROATIA |
| L.K.N. Arthrocentrum, s.r.o.  Na Valech 1  Hlucin, 748 01  CZECH REPUBLIC | Eticka komise IKEM a FTNsP  Videnska 800  Praha 4 Krc, 140 59  CZECH REPUBLIC |
| Revmatologicka ambulance  Petra Rezka 3  Praha 4, 140 00  CZECH REPUBLIC | Eticka komise IKEM a FTNsP  Videnska 800  Praha 4 Krc, 140 59  CZECH REPUBLIC |
| D. L. Pharma s.r.o.  Cejl 5  Brno, 60200  CZECH REPUBLIC  Fakultni nemocnice u sv. Anny v  Brne  Radiodiagnosticka klinika  Pekarska 53  Brno, 65691  CZECH REPUBLIC  Revmacentrum MUDr. Mostera,  s.r.o.  Mosnova 8  Brno - Zidenice, 615 00  CZECH REPUBLIC | Eticka komise IKEM a FTNsP  Videnska 800  Praha 4 Krc, 140 59  CZECH REPUBLIC |
| Oddzial Reumatologiczny,  Szpital im. Teodora Dunina  Samodzielny Publiczny Zespol  Opieki Zdrowotnej w Koscianie  Ul. Szpitalna 7  Koscian, 64-000  POLAND | Komisja Bioetyczna przy Okregowej  Izbie Lekarskiej w Gdansku  ul. Sniadeckich 33  Gdansk, 80-204  POLAND |
| Deaprtment of Internal Medicine  University Hospital Osijek  J.Huttlera 4  Osijek, 31000  CROATIA | Central Ethics Committee  Agency for Medicinal Products and  Medical Devices  Ksaverska c. 4  Zagreb, 10000  CROATIA |
| Jeffrey Alper M.D. Research  689 Ninth Street North  Naples, FL 34102  UNITED STATES | Quorum Institutional Review Board  Suite 1000  1601 Fifth Avenue  Seattle, WA 98101  UNITED STATES |
| Arthritis and Osteoporosis  Associates, LLP  5220 80th Street  Lubbock, TX 79424  UNITED STATES | Quorum Institutional Review Board  Suite 1000  1601 Fifth Avenue  Seattle, WA 98101  UNITED STATES |
| Arizona Arthritis and  Rheumatology Associates, PC  Suite F-150  10599 North Tatum Boulevard  Paradise Valley, AZ 85253  UNITED STATES  Arizona Arthritis and  Rheumatology Associates, PC  Suite 505  9305 West Thomas Road  Phoenix, AZ 85037  UNITED STATES  Arizona Arthritis and  Rheumatology Associates, PC  Suite 202  1500 South Dobson Road  Mesa, AZ 85202  UNITED STATES  Arizona Arthritis and  Rheumatology Associates, PC  Suite 204  5601 West Eugie Avenue  Glendale, AZ 85304  UNITED STATES | Quorum Institutional Review Board  Suite 1000  1601 Fifth Avenue  Seattle, WA 98101  UNITED STATES |
| Southwest Rheumatology, PA  Suite 615  18601 LBJ Freeway  Mesquite, TX 75150  UNITED STATES | Quorum Institutional Review Board  Suite 1000  1601 Fifth Avenue  Seattle, WA 98101  UNITED STATES |
| Rheuma Medicus -  Specjalistyczne Centrum  Reumatologii i Osteoporozy  Ul. Pruszkowska 6  Warszawa, 02-118  POLAND | Komisja Bioetyczna przy Okregowej  Izbie Lekarskiej w Gdansku  ul. Sniadeckich 33  Gdansk, 80-204  POLAND |
| NZOZ "NASZ LEKARZ"  Praktyka Grupowa Lekarzy  Rodzinnych z Przychodnia  Specjalistyczna  ul. Szczytna 20  Torun, 87-100  POLAND | Komisja Bioetyczna przy Okregowej  Izbie Lekarskiej w Gdansku  ul. Sniadeckich 33  Gdansk, 80-204  POLAND |
| Daegu Catholic University  Medical Center, Department of  Rheumatology  3056-6 Daemyung-4 dong  Nam-gu  Daegu, 705-718  KOREA, REPUBLIC OF | IRB of Daegu Catholic University  Medical Center  #202, Geumgang Villa, 991-8,  Daemyung-10 dong Nam-gu,  Daegu, 705-812  KOREA, REPUBLIC OF |
| Hospital Regional de Rancagua  Alameda #611  Rancagua, VI Region 2841959  CHILE | Comite Etico Cientifico  Servicio de Salud Metropolitano  Oriente  Avenida Salvador 364  Providencia, Santiago, RM 7500922  CHILE |
| DKTs Akta Medika EOOD,  Konsultativen kabinet po  Revmatologia  Diagnostic Consultative Center  ¿Akta Medika¿ EOOD  ul. Nikola Petkov 60  Sevlievo, 5400  BULGARIA | Ethics Committee for Multicenter  Trials  ul. "Damyan Gruev" 8  Sofia, 1303  BULGARIA  Komisiya po etika pri DKTs"Akta  Medika"/ Ethics Committee at DCC  "Akta Medika"  DKTs"Akta Medika" EOOD  Diagnostic Consultative Center  "Akta Medika" EOOD  ul. "Nikola Petkov" 60  Sevlievo, 5400  BULGARIA |
| FAE Innere Medizin /  Rheumatologie  Ludwig-Wucherer-Str. 10  Halle, 06108  GERMANY | Ethik-Kommission des Fachbereichs  Medizin der Justus-Liebig-  Universitaet Giessen  Gaffkystrasse 11 c  Giessen, 35385  GERMANY |
